# 

**Published:** 1893-07-29

**Authors:** 


					PRICE TWOPENCE. 1H W?
*o. 357, Vol. XIV.?July 29th, 1893. TL <?>
nUtfrtered at the General Pobi Oflioe as a Sew?P^^r for
trmonsUcion in t*" United KiTiurin"- ?r"1
WITH EXTRA NURSING SUPPLEMENT.
Per Annum, 8s. 6d., post free.
Monthly Parts, 6d.j post free, 8d.
Ditto per Annam, 8s., post free.
=? } Cs
No. 357. Vol. XIV. Saturday,
July 29th, 1893.
rT"wopgwcu.

				

